# Context-dependent codon partition models provide significant increases in model fit in atpB and rbcL protein-coding genes

**DOI:** 10.1186/1471-2148-11-145

**Published:** 2011-05-27

**Authors:** Guy Baele, Yves Van de Peer, Stijn Vansteelandt

**Affiliations:** 1 Department of Plant Systems Biology, VIB, B-9052 Ghent, Belgium; 2 Bioinformatics and Evolutionary Genomics, Department of Molecular Genetics, Ghent University, B-9052 Ghent, Belgium; 3 Department of Microbiology and Immunology, Rega Institute, K.U. Leuven, Kapucijnenvoer 33 blok I bus 7001, B-3000 Leuven, Belgium; 4 Department of Applied Mathematics and Computer Science, Ghent University, Krijgslaan 281 S9, B-9000 Ghent, Belgium

## Abstract

**Background:**

Accurate modelling of substitution processes in protein-coding sequences is often hampered by the computational burdens associated with full codon models. Lately, codon partition models have been proposed as a viable alternative, mimicking the substitution behaviour of codon models at a low computational cost. Such codon partition models however impose independent evolution of the different codon positions, which is overly restrictive from a biological point of view. Given that empirical research has provided indications of context-dependent substitution patterns at four-fold degenerate sites, we take those indications into account in this paper.

**Results:**

We present so-called context-dependent codon partition models to assess previous empirical claims that the evolution of four-fold degenerate sites is strongly dependent on the composition of its two flanking bases. To this end, we have estimated and compared various existing independent models, codon models, codon partition models and context-dependent codon partition models for the atpB and rbcL genes of the chloroplast genome, which are frequently used in plant systematics. Such context-dependent codon partition models employ a full dependency scheme for four-fold degenerate sites, whilst maintaining the independence assumption for the first and second codon positions.

**Conclusions:**

We show that, both in the atpB and rbcL alignments of a collection of land plants, these context-dependent codon partition models significantly improve model fit over existing codon partition models. Using Bayes factors based on thermodynamic integration, we show that in both datasets the same context-dependent codon partition model yields the largest increase in model fit compared to an independent evolutionary model. Context-dependent codon partition models hence perform closer to codon models, which remain the best performing models at a drastically increased computational cost, compared to codon partition models, but remain computationally interesting alternatives to codon models. Finally, we observe that the substitution patterns in both datasets are drastically different, leading to the conclusion that combined analysis of these two genes using a single model may not be advisable from a context-dependent point of view.

## Background

While the modelling of evolutionary processes in non-coding sequences has received much attention from a context-dependence point of view in the last two decades, the same cannot be said for modelling approaches for coding sequences, at least not in terms of developed model-based approaches. With the advent of new evolutionary models and drastic increases in computation power during the past decades, with desktop machines becoming more powerful and the advent of computer clusters with large amounts of processors (and processor cores) and a vast amount of memory, Maximum likelihood and Bayesian MCMC approaches now allow for very complex evolutionary models to be used in the analysis of large alignments.

Probabilistic modelling of sequence evolution has become the norm in phylogenetic inference, but complex evolutionary models are often not used in studies on molecular evolution, in part due to their increased computational burden but mainly due to the absence of such models in popular model testing tools [1]. Indeed, while researchers are widely adopting model selection techniques in phylogenetics in order to select the model that best fits their dataset, this may be problematic when complex evolutionary models are not included in popular model selection tools, such as Modeltest [2]. For example, in the case of analyzing protein-coding sequences, one needs to make sure to incorporate codon models as well as codon partition models in the model selection procedure.

Chloroplast genes, such as atpB and rbcL (the subjects of the analyses in this paper), are protein-coding genes that are often analyzed in concatenated alignments with non-coding sequences using independent nucleotide models (see e.g. [3,4]) or on their own using independent nucleotide models (see e.g. [5,6]). While the choice for an independent model of evolution, such as the general time-reversible model (GTR; [7]) in combination with varying rates across sites (RAS; e.g. [8]), is easily made in the case of non-coding sequences, such a choice is questionable when analyzing protein-coding sequences. Indeed, an inappropriate choice of evolutionary model can affect the outcome of any phylogenetic analysis (see [1] for an overview).

Models of codon substitution (i.e. full codon models) consider a codon triplet as the unit of evolution and can distinguish between synonymous and non-synonymous substitutions when analyzing protein-coding sequences [9]. This way, they are particularly effective in detecting signals of natural selection acting on the protein. An example of such a full codon model is the model of Goldman and Yang [10]. Even though the use of this model is computationally demanding due to the increased dimension of the substitution matrix, it is well suited for uncovering both recent and ancient divergences [9]. Another category of models to analyse protein-coding sequences are the so-called codon partition models, which use different nucleotide-based models to describe the evolutionary process at the different codon positions [1]. Codon partition models allow for different models of substitution for different partitions of the data. While full codon models - like the model of Goldman and Yang [10] - model biological reality more closely, codon partition models are much more computationally efficient [1].

In this paper, we aim to provide an overview of current codon partition models and present an extension of these models based upon previous empirical observations. Morton [11] studied the rbcL and ndhF genes from the chloroplast genome for signs of context dependence. Specifically, the author tested substitutions at four-fold degenerate sites for the existence of a correlation between neighbouring base composition and substitution bias and found that substitution bias, as measured by the proportion of transversions, is significantly different when neighbouring bases differ in their A+T contents. By performing chi-square tests for correlation on the numbers of observed transversions at four-fold degenerate sites and the A+T content of its neighbouring bases, Morton [11] found a significant difference in proportion of transversions in the three possible contexts (i.e. the three different levels of A+T content), both in an rbcL and ndhF dataset. These observations serve as the inspiration for this paper.

Here, we provide an overview of currently used codon partition models and assess their increase in model fit compared to the independent general-time reversible model [7] and the full codon model of Goldman and Yang [10]. Starting from the independent general-time reversible model of evolution, we build these codon partition models in a forward way, continually relaxing specific assumptions concerning evolution in protein-coding regions and calculating the difference in model fit each step of the way. We go on to extend these so-called site-independent codon partition models to context-dependent codon partition models by allowing for the evolution of the third codon position to depend upon the identities of its two immediate flanking positions (i.e. the second codon position of that same codon and the first codon position of the following codon) and by imposing specific Markov chains at the third codon position in the ancestral root sequence. We show that context-dependent evolution at the third codon position is a valuable feature to increase model fit in protein-coding sequences in general and that the properties of four-fold degenerate sites need to be taken into account when building a context-dependent codon partition model.

## Methods

### Data

**Figure 1:**
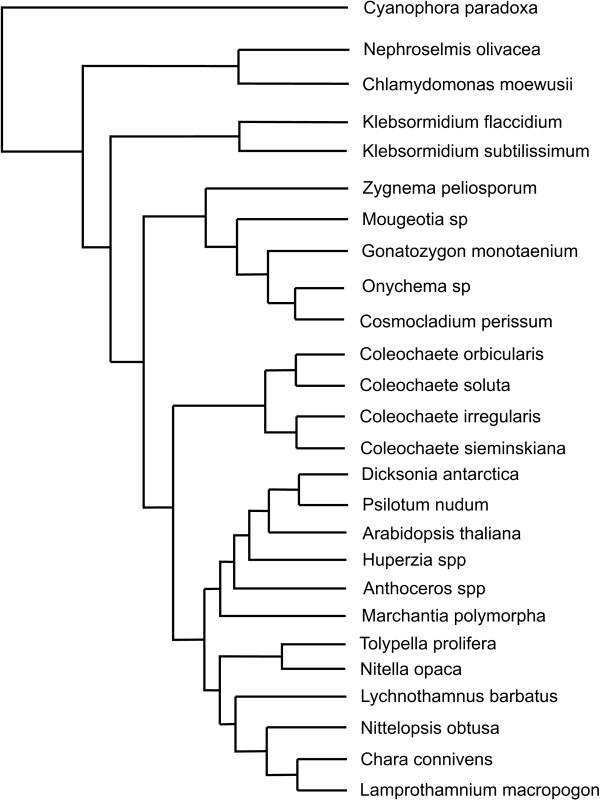
**Fixed consensus tree, based on the original tree inferred by Karol et al. **[3]**, branch lengths not drawn to scale**.

We have selected 26 sequences from the available 34 in the work of Karol et al. [3], for the atpB and rbcL protein-coding sequences. The following sequences were used: *Arabidopsis thaliana, Huperzia spp., Psilotum nudum, Dicksonia Antarctica, Anthoceros spp., Marchantia polymorpha, Chara connivens, Lamprothamnium macropogom, Lychnothamnus barbatus, Nittelopsis obtusa, Nitella opaca, Tolypella prolifera, Coleochaete orbicularis, Coleochaete solute, Coleochaete irregularis, Coleochaete sieminskiana, Onychonema sp., Cosmocladium perissum, Gonatozygon monotaenium, Zygnema peliosporum, Mougeotia sp., Klebsormidium flaccidium, Klebsormidium subtilissimum, Chlamydomonas spp., Nephroselmis olivacea and Cyanophora paradoxa *(as the outgroup). The sequence lengths (for each of the sequences) are 1203bp for the atpB dataset and 1206bp for the rbcL dataset. Our analyses used a fixed consensus tree for these 26 sequences, taken from the tree reported in Karol et al. [3], as can be seen in Figure 1 (branch lengths not drawn to scale).

### Site-independent evolutionary models

Site-independent models of evolution have been the main subject of many phylogenetic studies since the inception of the model of Jukes and Cantor [12]. Probably one of the most used evolutionary models is the general time-reversible model [7] with gamma-distributed rate heterogeneity (i.e. among-site rate variation, see e.g. [8,9]) and a proportion of invariant sites (although it is often argued that invariant sites are included in the category of lowest rates in the gamma distribution describing rate heterogeneity; see e.g. [9]), often denoted as GTR+Γ+I. For the combined alignment of the four genes (atpB (plastid), rbcL (plastid), nad5 (mitochondrial) and the small subunit (SSU) rRNA gene (nuclear)), Karol et al. [3] identified this model using a model selection approach as their model of choice for phylogenetic inference, although it is highly unlikely that this is the model that provides the highest fit to the data since more complex models for protein-coding sequences are often not present in popular packages for model selection (see e.g. [1]). We only consider two truly site-independent models in this paper, i.e. the GTR and the GTR+ Γ model. We have modelled the gamma-distributed rate heterogeneity using Yang's discrete approximation with 4 rate categories [8].

### Context-dependent (CD) models

Over the past two decades, various context-dependent models have been developed for analysing non-coding sequence (see [13] for a review). In previous work [13], we have developed a context-dependent model that employs a GTR model for each of the 16 possible neighbouring base combinations. We have introduced the notation "GTR16C" for this context-dependent model. A single set of site-independent base frequencies is used for all 16 GTR models, as well as to describe the ancestral root sequence distribution. This model drastically outperforms the independent GTR model, as well as the independent GTR model augmented with the assumption of gamma-distributed rates, for a dataset of primate ancestral repeats.

Even though protein-coding sequences can in principle not benefit from such models, we have tested the performance of our previously introduced context-dependent model [13] on both protein-coding datasets presented here. However, the main use of this context-dependent model lies in its introduction to the development of so-called context-dependent codon partition models, which will be discussed later in this paper.

### Full codon models

Goldman and Yang [10] developed one of the first codon-based evolutionary models, i.e. models that have codons as their states, incorporating biologically meaningful factors such as transition/transversion bias, variability of a gene and amino acid differences. Previous models describing the evolution of protein-coding genes in the field of phylogenetics worked either on the mononucleotide level in DNA sequences or on the amino acid level in protein sequences. While nucleotide models have 4 states and amino acid models have 20 states, the codon model of Goldman and Yang [10] has 61 states, i.e. the 61 sense codons, as it does not consider the 3 nonsense (stop) codons, given that mutations to or from stop codons can be assumed to drastically affect the structure and function of the protein and therefore will rarely survive.

Goldman and Yang [10] assume that mutations occur independently at the three codon positions and therefore allow only single-nucleotide substitutions to occur. Codons are also assumed to evolve independently from one another. The model multiplies rates of substitution involving a transition by a factor κ, which directly affects the ratio of transition and transversion substitutions. To account for selective restraints at the amino acid level, substitution rates are further modified by a multiplicative factor if the two codons code for different amino acids. To do so, the matrix of physicochemical distances between the 20 amino acids, as composed by Grantham [10], is used. In the implementation of this model, which we denote "GY94", we have used the averages of the observed codon frequencies, as suggested by Goldman and Yang [10]. We also allow for among-codon rate variation and denote such a model as "GY94+Γ".

### Codon Partition (CP) models

Codon partition (CP) models are nucleotide models that accommodate the differences in the evolutionary dynamics at the three codon positions (see e.g. [9,14]). For example, Yang [14] takes into account the nucleotide frequency bias, the substitution rate bias and the difference in the extent of rate variation among the three codon positions. While Yang [14] only used the HKY evolutionary model [15] in his analyses, Shapiro et al. [1] also included the GTR model. In their comparative study of suitable models for protein-coding sequence, Shapiro et al. [1] found that for only 2 out of 283 multiple sequence alignments, the GTR+Γ+I model was the best model and for only 1 of them, GTR+ Γ was the model of choice. Likewise, the HKY+I model was only favoured in 1 out of the 283 alignments. As shown by Shapiro et al. [1], codon partition (CP) models are biologically motivated and are a computationally feasible alternative to codon-based models for the analysis of protein-coding sequences, since the dimensions of the evolutionary matrices do not increase.

We here describe currently used codon partition models (along with their notation in this paper) but restrict ourselves to those models that use the general time-reversible (GTR) evolutionary model. We do not use nor describe models of invariable sites plus gamma-distributed rates (so-called ''I+ Γ'' models), given the strong correlation between the proportion of invariable sites and the gamma shape parameter (see e.g. [9]). All the codon partition models discussed in this section assume that the different codon positions evolve independently from one another.

A first series of models, denoted GTR_112 _and GTR_123_, employ different general time-reversible models depending on codon position. While the GTR_112 _model groups the first and second codon position together and hence uses 2 models (10 free parameters), the GTR_123 _model considers each codon positions separately and hence uses 3 models (15 free parameters). This is also referred to as the accommodation of substitution rate bias.

These two models can be linked with one common set of base frequencies (3 free parameters) in which case we simply denote them as GTR_112 _and GTR_123_. However, these two models can also be combined with 2 sets of base frequencies (6 free parameters) for the GTR_112 _model and 3 sets of base frequencies (9 free parameters) for the GTR_123 _model and we denote such models with a "+F" notation, i.e. GTR_112_+F_112 _and GTR_123_+F_123_. This is also referred to as the accommodation of nucleotide frequency bias.

An assumption concerning the evolutionary behaviour of sites in an alignment that typically greatly improves model fit as well as phylogenetic tree reconstruction is that of gamma-distributed rate-heterogeneity, although typically used in non-coding sequence data. In the case of a discrete approximation to rate heterogeneity for all positions, we use the typical "+Γ" notation. Modelling rate heterogeneity requires only one extra parameter. However, given the difference in evolutionary dynamics across codon positions, it may be important to allow for the extent of rate variation to vary across sites. This means using two independent gamma distributions when the first and second codon positions are grouped together and using three independent gamma distributions when the three codon positions are modelled separately. We denote these distributions, which require two and three extra parameters, respectively with a "+Γ_112_" and a "+Γ_123_" notation.

When simply allowing for different distributions for the rate heterogeneity at different codon positions, it is assumed that the mean rate for each of these distributions is 1. As this may turn out to be unrealistic, so-called rate ratios can be added to allow for different mean rates in the gamma distributions (i.e. to allow for variable mutation rates among the different codon positions). We have used the random-rates approach presented in the work of Burgess and Yang [16] to accommodate variable mutation rates among codon positions (i.e. the average rate is fixed at 1). In accordance with the work of Shapiro et al. [1], we use the notations "+CP_112_" and "+CP_123_" to indicate the models with variable mutation rates among codon positions.

### Context-Dependent Codon Partition (CDCP) models

While the current codon partition (CP) models (see e.g. [1,9,14]) allow for different evolutionary dynamics at the three codon positions, such models do not incorporate dependencies between the different codon partitions (i.e. each codon position is still assumed to evolve independently). Allowing for such dependencies is a particular strength of full codon models. In this paper, we provide a series of possible extensions for current codon partition models, based on the assumption that codon positions do not evolve independently from one another. As stated in the introduction, our context-dependent modelling assumptions are inspired by the work of Morton [11], who detected a significant correlation between the number of transversions at four-fold degenerate sites and the A+T content of the two immediate flanking bases.

As a first attempt, we assume context-dependent evolution of the third codon position on its two immediate flanking bases (i.e. the second codon position of the same codon and the first codon position of the succeeding codon). The first and second codon positions however are still assumed to evolve independently from one another. We model this dependence by assuming that evolution at the third codon position occurs according to our full context-dependent GTR16C model [13]. This means that for the third codon position, 16 GTR models (but each employing the same (single) set of base frequencies) will be estimated, one for each neighbouring base combination, resulting in 80 free evolutionary parameters to be estimated for the third codon position (compared to 5 free evolutionary parameters for the first codon position and the second codon position). We use the notation "+CD_16_" for this assumption.

One of the main conclusions of the study of Morton [11] is that the number of transversions at the third codon position are correlated with the A+T content of its two immediate flanking bases. To incorporate this finding, we have adapted our full context-dependent model (GTR16C; see [13]) to only combine 3 GTR models (or 15 free parameters) for the third codon position: 1 for an A+T content of 0 (i.e. none of the two immediate flanking bases is either A or T), 1 for an A+T content of 1 (i.e. exactly one of the two immediate flanking bases is either A or T) and 1 for an A+T content of 2 (i.e. both immediate flanking bases are either A or T). We use the notation "+CD_3_" for this assumption. Note that both the first and second codon positions are still allowed to evolve independently.

### Four-fold degenerate sites

A position of a codon is said to be a four-fold degenerate site if any nucleotide at this position specifies the same amino acid. For example, the third position of the glycine codons (GGA, GGG, GGC, GGU) is a four-fold degenerate site, because all nucleotide substitutions at this site are synonymous, i.e. they do not change the amino acid. Only the third positions of some codons may be fourfold degenerate. The following amino acids have four-fold degenerate codon positions: alanine (GCX), arginine (CGX), glycine (GGX), leucine (CUX), praline (CCX), threonine (ACX), serine (UCX) and valine (GUX).

Here, we expand the context-dependent codon partition model introduced in the previous section in that the context-dependent model at the third codon position is only used for the four-fold degenerate sites, as identified at the start of each branch. Non four-fold degenerate sites are assumed to evolve according to a separate site-independent general time-reversible model, which shares the same (single) set of base frequencies as the context-dependent model at the four-fold degenerate sites. When assuming the full context-dependent model at the third codon position, we denote this model "+FF_16_"; when using the context-dependent model at the third codon position that is aimed at modelling a correlation to the A+T context of its two immediate flanking bases, we denote the model as "+FF_3_".

### Ancestral root sequence distribution

In previous work on context-dependent models for non-coding sequences, we have extensively discussed the importance of an adequate ancestral root sequence distribution to estimate the evolutionary parameters of context-dependent models [17]. Indeed, while the context-dependent models presented in the two previous sections impose a dependency scheme at the third codon position across the underlying tree, it does not in any way impose a dependency pattern at the ancestral root of the underlying phylogenetic tree. To ensure a dependency scheme across the entire underlying phylogenetic tree, we therefore allow for different ancestral root distributions for the third codon position.

The standard approach would be to use the values of the base frequencies describing the contents of the third codon position as the prior probability for the states of the third codon position (see e.g. [18]), which assumes independence at the ancestral root sequence. An approach which we have shown to be practical when modelling context-dependent evolution in non-coding sequences [17], is to assume a zero-order Markov chain (i.e. a separate set of independent base frequencies *π*_*ROOT *_= {*π*_*A*_, *π*_*C*_, *π*_*G*_, *π*_*T*_}) to describe the ancestral root distribution at the third codon position. This approach hence requires merely 3 additional free parameters.

An extension of this approach is to use a first-order Markov chain to allow for dependence of the third codon position on the second codon position (of the same codon) in the ancestral root sequence, which requires four independent sets of base frequencies *π*_*ROOT *_= {*π*_*X|A*_, *π*_*X|C*_, *π*_*X|G*_, *π*_*X|T*_}, with *X *∈ {*A,C,G,T*} the identity of the site at the second codon position of the same codon. One could also choose to model a first-order Markov chain in the opposite direction, i.e. where the identity of the third codon position would depend upon the identity of the first codon position of the succeeding codon. However, it is realistic to expect that this approach will be less optimal in terms of model fit, so we did not attempt to include this dependency scheme. Note that our notation for the distribution at the root sequence differs from the typical notation for conditional probabilities in that we put the conditional part before the 'pipe' symbol ('|').

A final approach to model context-dependence at the third codon position in the ancestral root sequence is to allow its identity to depend upon the identity of its two immediate flanking bases, i.e. the identity of the second codon position of the same codon and the identity of the first codon position of the succeeding codon. We model this "second-order" dependence through 16 sets of base frequencies *π*_*ROOT *_= {*π*_*X|A|Y*_, *π*_*X|C|Y*_, *π*_*X|G|Y*_, *π*_*X|T|Y*_}, with *X *∈ {*A, C, G, T*} the identity of the site at the second codon position of the same codon and *Y *∈ {*A, C, G, T*} the identity of the site at the first codon position of the succeeding codon.

### Bayesian Markov Chain Monte Carlo using data augmentation

Bayesian inference of phylogeny is based on a quantity called the posterior probability function of a tree, in the same way as maximum-likelihood inference is based on the likelihood function. While the posterior probability is generally tedious to calculate, simulating from it is relatively easy through the use of Markov chain Monte Carlo (MCMC) methods ([19,20]). Relaxing the assumption of independent evolution leads to more parameter-rich evolutionary models and computational difficulties, which we handle via a data augmentation scheme [21]. As previously discussed [22], consistency problems may arise with such high-dimensional models, along with potential computational burdens. In view of this, a model-selection approach should be used that penalizes the addition of extra parameters unless there is a sufficiently impressive improvement in fit between model and data [22]. To compare the different assumptions concerning the root distribution, we have calculated (log) Bayes Factors [23]. Log Bayes Factors are typically divided into 4 categories depending on their value: from 0 to 1, indicating nothing worth reporting; from 1 to 3, indicating positive evidence of one model over the other; from 3 to 5, indicating strong evidence of one model over the other; and larger than 5, indicating very strong evidence of one model over the other. We have chosen to calculate Bayes Factors using thermodynamic integration [24], since the traditional harmonic mean estimator of the marginal likelihood systematically favours parameter-rich models and is hence unfit to compare these complex context-dependent models. A detailed discussion of the data augmentation approach in our proposed Bayesian Markov chain Monte Carlo approach and in the thermodynamic integration framework for model comparison can be found in previous work [13,25].

### Prior Distributions

Let *T *be the set of branch lengths with *t_b _*(*t_b_*≥0) one arbitrary branch length and μ a hyperparameter in the prior for *t_b _*in *T *(see e.g. [26]). The following prior distributions *q *(·) are chosen for our analysis, with Γ(·) the Gamma function. Dirichlet priors (which are uninformative priors) assign densities to groups of parameters that measure proportions (i.e., parameters that must sum to 1). For each set of base frequencies (which we here denote *π*_1,2,3_) that describes the nucleotide composition of a codon position (and also the ancestral root distribution), the following prior distribution is assumed:

For each set of model frequencies of which the ancestral root sequence is composed, the following prior distribution is assumed:

As mentioned earlier, we have used the random-rates approach presented in the work of Burgess and Yang [16] to accommodate variable mutation rates among codon positions. Let the rate at codon position *i *be *r_i_*, with *i *= 1,2,3. To avoid overparameterization, the average rate is fixed at one:  and a Dirichlet prior is assigned on the variables . For additional information on this prior assignment, we refer to the work of Burgess and Yang [16].

For each codon position, the parameter *α_i_*, with *i *= 1,2,3, describing the among-site rate variation gamma-distribution at that codon position, is assumed to follow a uniformly distributed prior between 0 and 50.

For the model parameters of each context (i.e. neighbouring base combination) independently, the following prior distribution is assumed (see e.g. [27]):

Further, branch lengths are assumed i.i.d. given *μ*:

and

## Results

### atpB dataset

**Figure 2:**
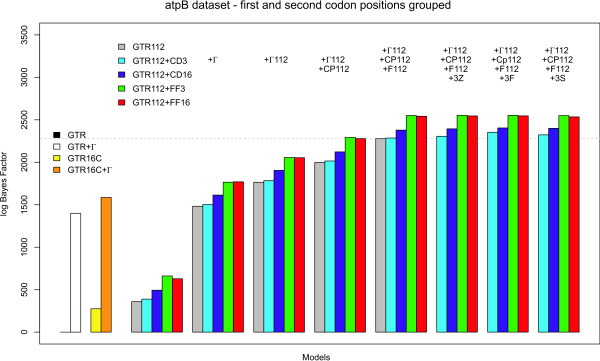
**atpB dataset: Comparison of all models presented in this paper to the GTR model, when grouping the first and second codon positions together**. The performance of "traditional" codon partition models (up to the dotted horizontal line) can be improved significantly by assuming context-dependent evolution at the third codon position. As can be seen from this figure, such context-dependent codon partition models systematically outperform the (independent) codon partition models in terms of model fit.

**Figure 3:**
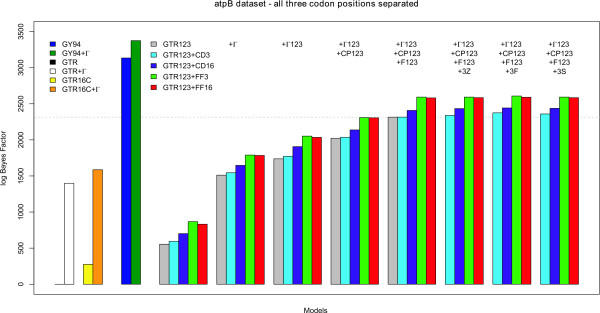
**atpB dataset: Comparison of all models presented in this paper to the GTR model, when treating each codon position separately**. The performance of "traditional" codon partition models (up to the dotted horizontal line) can be improved significantly by assuming context-dependent evolution at the third codon position. As is confirmed in this figure, such context-dependent codon partition models systematically outperform the (independent) codon partition models in terms of model fit. Further, the models that assume that first and second codon positions evolve according to a separate evolutionary model (in this figure) systematically outperform those models that treat first and second codon positions identically (from an evolutionary perspective).

Using the model-switch thermodynamic integration scheme, we have compared all site-independent, codon partition and context-dependent codon partition model to the independent general time-reversible model (GTR). Figure 2 shows all calculated bidirectional mean log Bayes Factors for those evolutionary models that group first and second codon positions, while Figure 3 shows the results for those evolutionary models that consider each codon position separately (annealing and melting thermodynamic integration results can be found in Tables S1, S2 and S3 in Additional file 1). The presented models are constructed from the bottom up, i.e. from left to right in Figures 2 and 3, each time an additional evolutionary assumption is relaxed with respect to the models (i.e. additional parameters are added). For matters of comparison, the left-hand side of Figures 2 and 3 show the performances of 4 models that can be used in the analysis of non-coding sequences: GTR (black line; mean log Bayes Factor of 0), GTR+Γ (white bar; using 4 discrete rate categories; mean log Bayes Factor of 1398.01); GTR16C (yellow bar; mean log Bayes Factor of 275.08); and GTR16C+Γ (orange bar; mean log Bayes Factor of 1585.80).

The overall trend in Figures 2 and 3 is identical, albeit that the results presented in Figure 3 (where each codon position is treated separately) systematically outperform those presented in Figure 2 (where the first and second codon positions are grouped together). The basic model in Figure 2 is hence the GTR_112 _model, while the basic model in Figure 3 is the GTR_123 _model. To start the various model comparisons, both the GTR_112 _and GTR_123 _models are augmented with the "+CD_3_", "+CD_16_", "+FF_3_" and "+FF_16_" context-dependent assumptions. In both Figures 2 and 3 it can clearly be seen that the context-dependent substitution patterns for the third codon position increase model fit substantially, even without accommodating additional evolutionary effects such as nucleotide frequency bias or assuming among-site rate variation for each codon position (see Tables S1, S2 and S3 in Additional file 1 for numerical results). These context-dependent codon partition models continue to outperform the traditional codon partition models with the inclusion of all the evolutionary assumptions discussed in the Methods section.

Allowing for among-site variation ("+Γ" in both Figures 2 and 3; using 4 discrete rate categories, as is conventional when analyzing non-coding sequences) yields the largest increase in model fit. As it makes more sense in coding sequences to assume a specific pattern of among-site rate variation for either the first and second codon positions grouped together ("+Γ_112_" in Figure 2) or for each codon position separately ("+Γ_123_" in Figure 3), the corresponding models clearly outperform a model which imposes 1 general pattern of among-site rate variation for all sites. To relax the restriction that each codon position has a mean evolutionary rate of 1.0, which is deemed unrealistic, this is usually accompanied by a different mean evolutionary rate per codon position ("+CP_112_" in Figure 2 and "+CP_123_" in Figure 3).

Given that up to this point, one single set of base frequencies has been used for the different codon positions, we have relaxed this assumption and have modelled the so-called nucleotide frequency bias ("+F_112_" in Figure 2 and "+F_123_" in Figure 3). For both the GTR_112 _and GTR_123 _models without the assumption of context-dependence for the third codon position, this latest inclusion marks the optimal model fit as presented in the literature. We have marked the corresponding increase in model fit in both Figures 2 and 3 by a dotted horizontal line. Assuming context-dependence for all third codon positions on the A+T context of the two immediate flanking bases (GTR_112_+Γ_112_+CP_112_+F_112_+CD_3 _and GTR_123_+Γ_123_+CP_123_+F_123_+CD_3_) does not yield an increase over its independence counterpart (GTR_112_+Γ_112_+CP_112_+F_112 _and GTR_123_+Γ_123_+CP_123_+F_123_). All other dependency patterns at the third codon position have however resulted in increases vis-à-vis the current state-of-the-art codon partition models, with the GTR_123_+Γ_123_+CP_123_+F_123_+FF_3 _model yielding the largest increase in model fit.

Of the different ancestral root distributions proposed to model a dependency scheme at the third codon position, the first-order Markov chain (i.e., model GTR_123_+Γ_123_+CP_123_+F_123_+FF_3_+3F) offers the largest improvement in model fit across the different model comparisons, followed by the GTR_123_+Γ_123_+CP_123_+F_123_+FF_16_+3F model. The same pattern can be seen when grouping first and second codon positions together, but these models are consistently outperformed by those models that treat each codon position separately.

Figure 3 also shows the performance of the full codon model of Goldman and Yang [10]. The GY94 model yields an increase in model fit vis-à-vis the GTR model of 3134.49 log units (annealing 95% CI: [3096.16; 3130.80]; melting 95% CI: [3136.83; 3174.19]) and the GY94+Γ model (annealing 95% CI: [3277.70; 3331.63]; melting 95% CI: [3416.64; 3472.83]) an increase of 3374.70 log units. We have included this result for the sake of comparison, as these models mimic full codon models. We thus find that the proposed context-dependent codon partition models improve upon the existing codon partition models and partially close the gap with full codon models. Note, however, that this is not the main point of such a comparison, given that the presented models include dependencies across the codon boundaries and are hence not exclusively aimed towards mimicking codon models more closely.

**Figure 4:**
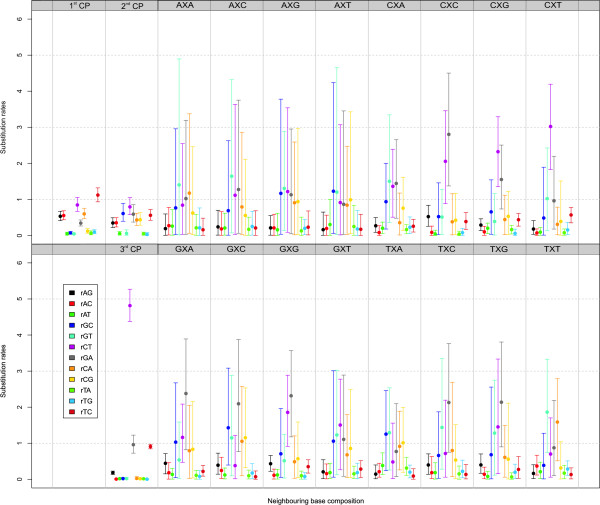
**atpB dataset: Estimated substitution patterns for the independent evolutionary models at first, second and third codon positions and the context-dependent evolutionary model at the third codon position for the GTR_123_+Γ_123_+CP_123_+F_123_+FF_16_+3F model**. At the left of this figure, i.e. the first two columns, the independent model estimates for first (1^st ^CP), second (2^nd ^CP) and third (3^rd ^CP) codon positions are shown. At the right of the figure (columns 3 through 10), the context-dependent estimates for the third codon position are shown for each evolutionary context, i.e. each neighbouring base combination. In the figure, the substitution parameter types (i.e. rAG, rAC, ..., rTC) each have a unique colour, as indicated in the legend panel within the figure, and the same colour is used for every model (context-dependent or not).

As we have shown in previous work [25], parameter clustering may drastically increase model fit of a given context-dependent evolutionary model. While this is not the aim of this study, it is thus quite realistic that clustering several parameters together will result in the GTR_123_+Γ_123_+CP_123_+F_123_+FF_16_+3F model being the optimal codon partition model for the atpB dataset. This is why we go on to discuss the parameter estimates for this model. The parameter estimates shown in Figure 4 support the model building approach we have taken in this paper. For instance, the evolutionary estimates for those third codon position that are four-fold degenerate sites, are clearly different from those of the first two codon positions, which are clearly different from one another as well (for example, the rGC, rCG and rTC estimates), justifying the use of a separate evolutionary model for all three codon positions. Particularly interesting is the increased rCT substitution rate at those third codon positions that are not four-fold degenerate sites.

The context-dependent substitution rates, which are grouped by the identity of the left neighbouring base, form the most interesting aspect of Figure 4. Note that, given the small size of the (single-gene) alignment and the multitude of substitution rates to be estimated, the 95% credibility intervals are quite wide. Further, it is important to assess whether there are bidirectional dependencies on the third codon position, i.e. whether there is an influence of the identities of both left and right neighbouring bases on the substitution rate. We discuss the context-dependent substitution rates for the four-fold degenerate sites, as grouped by the identity of their left neighbouring base, in Additional file 1. Important to notice is that the relationship between these substitution rates and the immediate neighbouring bases extends well beyond an influence of the A+T context of those bases, as proposed by Morton [11].

Table 1 shows drastic differences in the nucleotide frequency estimates for the three codon positions, illustrating the importance of not grouping first and second codon positions together, as shown by the calculated (log) Bayes Factors in Tables S1 through S3 in Additional file 1. Further, there are clearly large differences in the degree of among-site rate variation across codon positions. All three gamma distributions are L-shaped, meaning that for each codon position, most sites have very low substitution rates or are virtually invariable, while a few sites exist with very high rates [8]. While the first codon positions evolve on average twice as fast as the second codon positions, the mean rate for the third codon position is drastically elevated compared to the first and second codon positions, as indicated by the estimates for the relative rates in Table 1.

**Table 1:** atpB dataset: estimates per codon position for the nucleotide frequencies, among-site rate variation distribution and relative rates (for the GTR_123_+Γ_123_+CP_123_+F_123_+FF_16_+3F model).

	Frequencies					
**CP**	**A**	**C**	**G**	**T**		**CP_123_**

1	0.2396 [0.21; 0.28]	0.2216 [0.19; 0.26]	0.3717 [0.33; 0.42]	0.1671 [0.14; 0.20]	0.1579 [0.11; 0.24]	0.1066 [0.08; 0.14]
2	0.2804 [0.24; 0.33]	0.2298 [0.19; 0.27]	0.1656 [0.13; 0.20]	0.3242 [0.28; 0.38]	0.0946 [0.01; 0.15]	0.0512 [0.04; 0.07]
3	0.3784 [0.35; 0.41]	0.0875 [0.08; 0.10]	0.0707 [0.06; 0.08]	0.4634 [0.43; 0.50]	0.8716 [0.73; 1.04]	2.8422 [2.80; 2.88]

Shown are mean estimates of 100.000 MCMC iterations, discarding the first 20.000 iterations as the burn-in, and the corresponding 95% credibility intervals. The values in the table clearly illustrate the different substitution dynamics at the three codon positions.

Table 2 shows the estimates of the first-order Markov chain imposed on the third codon position in the ancestral root sequence for the atpB dataset. While the mean values of the estimates reveal large differences in the first-order estimates, they are often accompanied by very wide 95% credibility intervals, which is why this ancestral root distribution at the third codon position offers only a small improvement on average over the other assumptions (zero-order Markov chain and dependence on both neighbouring bases; see Table S3 in Additional file 1).

**Table 2:** atpB dataset: estimates for the first-order Markov chain distribution at the ancestral root sequence (for the GTR_123_+Γ_123_+CP_123_+F_123_+FF_16_+3F model).

	Root			
**X**	***π_X|A_***	***π_X|C_***	***π_X|G_***	***π_X|T_***

A	0.5049 [0.38; 0.61]	0.4089 [0.28; 0.52]	0.0382 [0.00; 0.13]	0.0480 [0.00; 0.16]
C	0.1018 [0.00; 0.31]	0.2481 [0.01; 0.75]	0.1993 [0.01; 0.55]	0.4507 [0.06; 0.79]
G	0.0402 [0.00; 0.15]	0.1610 [0.00; 0.52]	0.1088 [0.01; 0.40]	0.6899 [0.16; 0.94]
T	0.1010 [0.01; 0.25]	0.3925 [0.22; 0.54]	0.4490 [0.29; 0.59]	0.0575 [0.00; 0.20]

Shown are mean estimates of 100.000 MCMC iterations, discarding the first 20.000 iterations as the burn-in, and the corresponding 95% credibility intervals. Despite the wide 95% credibility intervals, the estimates show why such a first-order dependence is useful.

### rbcL dataset

**Figure 5:**
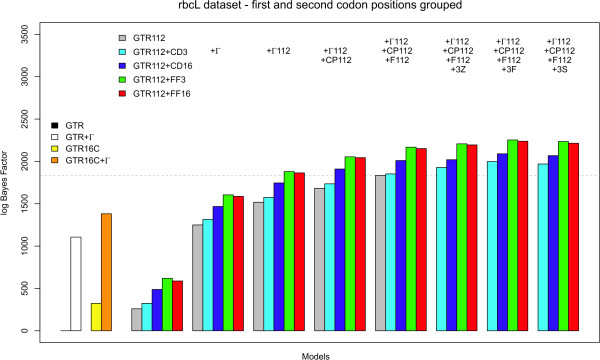
**rbcL dataset: Comparison of all models presented in this paper to the GTR model, when grouping the first and second codon positions together**. The performance of "traditional" codon partition models (up to the dotted horizontal line) can be improved significantly by assuming context-dependent evolution at the third codon position. As can be seen from this figure, such context-dependent codon partition models systematically outperform the (independent) codon partition models in terms of model fit.

**Figure 6:**
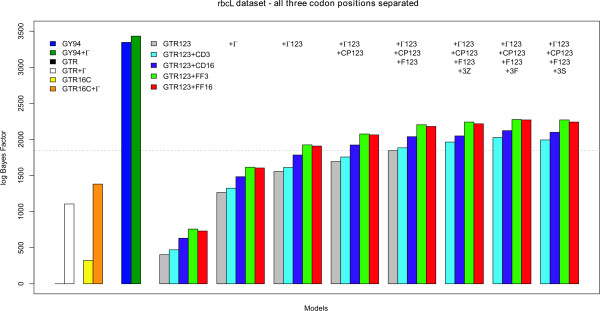
**rbcL dataset: Comparison of all models presented in this paper to the GTR model, when treating each codon position separately**. The performance of "traditional" codon partition models (up to the dotted horizontal line) can be improved significantly by assuming context-dependent evolution at the third codon position. As is confirmed in this figure, such context-dependent codon partition models systematically outperform the (independent) codon partition models in terms of model fit. Further, the models that assume that first and second codon positions evolve according to a separately evolutionary model (in this figure) systematically outperform those models that treat first and second codon positions identically (from an evolutionary perspective).

As for the atpB dataset, we have compared all codon partition and context-dependent codon partition model to the independent general time-reversible model using the model-switch thermodynamic integration scheme. Figure 5 shows all the calculated bidirectional mean log Bayes Factors for those evolutionary models that group first and second codon positions, while Figure 6 shows the results for those evolutionary models that consider each codon position separately (annealing and melting thermodynamic integration results can be found in Tables S4, S5 and S6 in Additional file 1). For matters of comparison, the left-hand side of Figures 5 and 6 show the performances of 4 models that can be used in the analysis of non-coding sequences: GTR (black line; mean log Bayes Factor of 0), GTR+Γ (white bar; using 4 discrete rate categories; mean log Bayes Factor of 1106.06); GTR16C (yellow bar; mean log Bayes Factor of 323.17); and GTR16C+Γ (orange bar; mean log Bayes Factor of 1381.77).

The overall trend in Figures 5 and 6 is identical to that in Figures 2 and 3. Comparing Tables S1 through S6 in Additional file 1 reveals that not only the hierarchy of the models is identical, but also that the exact same model is preferred for the rbcL dataset as for the atpB dataset. However, despite the fact that the rbcL alignment is only 3 sites shorter than the atpB alignment (i.e. 0.25% shorter), there are large differences in the calculated log Bayes Factors across the models evaluated. For example, the log Bayes Factor for the GTR_123_+Γ_123_+CP_123_+F_123_+FF_16_+3F model compared to the GTR model for the atpB datasets is 313 log units higher than the corresponding log Bayes Factor for the rbcL dataset. Hence, as was the case for the atpB dataset, it can be seen from Figures 5 and 6 that the context-dependent substitution patterns for the third codon position increase model fit substantially, even without accommodating additional evolutionary effects such as nucleotide frequency bias or among-site rate variation for each codon position (see Tables S4, S5 and S6 in Additional file 1 for numerical results) and that these context-dependent codon partition models outperform traditional codon partition models.

Figure 6 also shows the performance of the full codon model of Goldman and Yang [10], which outperforms all other models as was the case for the atpB dataset. The GY94 model yields an increase in model fit vis-à-vis the GTR model of 3134.49 log units (annealing 95% CI: [3309.51; 3339.88]; melting 95% CI: [3349.88; 3382.22]) and the GY94+Γ model (annealing 95% CI: [3356.12; 3408.43]; melting 95% CI: [3459.73; 3505.79]) an increase of 3374.70 log units.

**Figure 7:**
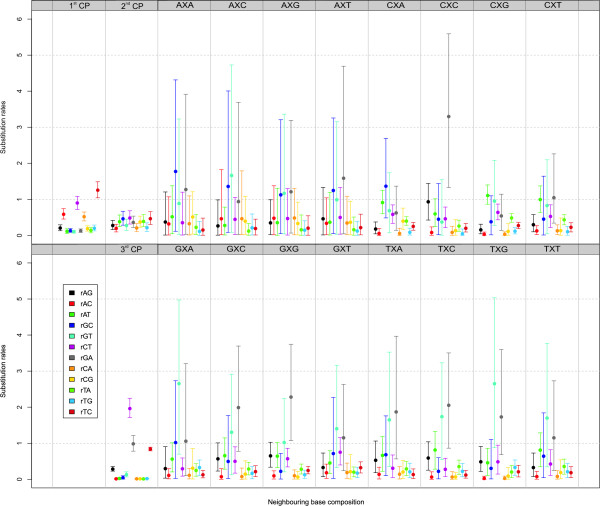
**rbcL dataset: Estimated substitution patterns for the independent evolutionary models at first, second and third codon positions and the context-dependent evolutionary model at the third codon position for the GTR_123_+Γ_123_+CP_123_+F_123_+FF_16_+3F model**. At the left of this figure, i.e. the first two columns, the independent model estimates for first (1^st ^CP), second (2^nd ^CP) and third (3^rd ^CP) codon positions are shown. At the right of the figure (columns 3 through 10), the context-dependent estimates for the third codon position are shown for each evolutionary context, i.e. each neighbouring base combination. In the figure, the substitution parameter types (i.e. rAG, rAC, ..., rTC) each have a unique colour, as indicated in the legend panel within the figure, and the same colour is used for every model (context-dependent or not).

The parameter estimates for the optimal GTR_123_+Γ_123_+CP_123_+F_123_+FF_16_+3F model, which are shown in Figure 7, are discussed in Additional file 1. Important to notice is once again that the relationship between these substitution rates and the immediate neighbouring bases extends well beyond an influence of the A+T context of those bases, as proposed by Morton [11]. In short, compared to Figure 4 which contains the context-dependent estimates for the atpB dataset, it can be concluded from Figure 7 that the overall rCT substitution rate is drastically lower in the rbcL dataset across all the neighbouring base combinations (as was the case for the non four-fold degenerate sites); the 95% credibility intervals are much smaller. Moreover, the rCT estimates do not differ all that much depending on the neighbouring base combinations, as opposed to the atpB dataset. Further, the rCA and rCG substitution rates are also lower than in the atpB dataset and have much smaller 95% credibility intervals as well. On the other hand, the 95% credibility intervals for the rAG and rAT estimates are larger than in the atpB dataset. In short, the main differences in terms of the context-dependent substitution rates at the four-fold degenerate sites can be found in the rGC, rGT and rGA parameters.

The observation that the overall substitution behaviour as well as the context-dependent substitution patterns at the fourfold degenerate sites differs between the atpB and the rbcL genes is not surprising as Morton [11] already reported that rbcL is the exceptional case in the chloroplast genes in terms of the relationship between context and substitution bias. As a possible explanation for this observation, Morton [11] suggested that a more complex context dependency exists than the relationship between substitution bias and A+T context that the author has studied. However, given that we have estimated more complex context-dependent substitution patterns at the fourfold degenerate sites, in that we don't refrain ourselves from only distinguishing between transitions and transversions, we still observe clearly different substitution behaviour between the two datasets. Additional datasets, such as the ndhF, psbA, matK, atpA, ..., genes, should be studied to further clarify the substitution patterns in the chloroplast genes. Unfortunately, these genes have not yet been sequenced for the majority of the organisms used in this paper.

As for the atpB dataset, Table 3 shows drastic differences in the nucleotide frequency estimates for the three codon positions, which is reflected in the higher log Bayes Factors for those models that treat all three codon positions separately (see Tables S4 through S6 in Additional file 1). A notable difference between the two datasets is the underrepresentation of cytosine at the third codon position in the atpB dataset, which is not the case in the rbcL dataset. Further, while the among-site rate distribution parameters for the first and second codon positions are quite similar for both datasets, this is not the case for the third codon position, where the gamma distribution parameter is three times higher for the rbcL dataset. Hence, only the gamma distributions for the first and second codon positions are L-shaped, meaning that for each codon position, most sites have very low substitution rates or are virtually invariable, while a few sites exist with very high rates. The gamma distribution for the third codon position is bell-shaped, meaning that most sites have intermediate rates while few sites have very low or very high rates [8]. The relative rate estimates for the rbcL dataset also show some differences compared to the estimates of the atpB dataset. The mean rates of the first and second codon positions are two to three times higher than in the atpB dataset, with the mean rate at the third codon position dropping about 10 percent.

**Table 3:** rbcL dataset: estimates per codon position for the nucleotide frequencies, among-site rate variation distribution and relative rates (for the GTR_123_+Γ_123_+CP_123_+F_123_+FF_16_+3F model).

	Frequencies					
**CP**	**A**	**C**	**G**	**T**		**CP_123_**

1	0.2202 [0.19; 0.26]	0.2484 [0.22; 0.28]	0.3536 [0.31; 0.40]	0.1778 [0.15; 0.21]	0.1948 [0.15; 0.26]	0.2802 [0.23; 0.34]
2	0.2712 [0.22; 0.32]	0.2558 [0.20; 0.31]	0.2071 [0.16; 0.26]	0.2660 [0.22; 0.32]	0.0465 [0.00; 0.11]	0.1212 [0.09; 0.16]
3	0.2200 [0.18; 0.26]	0.2152 [0.19; 0.25]	0.0634 [0.05; 0.08]	0.5014 [0.46; 0.54]	2.6956 [1.92; 3.84]	2.5986 [2.53; 2.66]

Shown are mean estimates of 100.000 MCMC iterations, discarding the first 20.000 iterations as the burn-in, and the corresponding 95% credibility intervals. The values in the table clearly illustrate the different substitution dynamics at the three codon positions. There are many differences with the estimates of the atpB dataset, shown in Table 1. The most notable differences are the among-site rate variation distribution parameter and the nucleotide frequencies for the third codon position.

Table 4 shows the estimates of the first-order Markov chain imposed on the third codon position in the ancestral root sequence for the rbcL dataset. The mean values of the estimates are once again accompanied by wide 95% credibility intervals, although not to the extent of the atpB dataset. While the distribution of frequencies is quite similar when the preceding base is adenine, for the other preceding bases this isn't the case, with the largest difference occurring when the preceding base is cytosine.

**Table 4:** rbcL dataset: estimates for the first-order Markov chain distribution at the ancestral root sequence (for the GTR_123_+Γ_123_+CP_123_+F_123_+FF_16_+3F model).

	Root			
**X**	***π_X|A_***	***π_X|C_***	***π_X|G_***	***π_X|T_***

A	0.4225 [0.33; 0.51]	0.5464 [0.46; 0.64]	0.0159 [0.00; 0.06]	0.0151 [0.00; 0.06]
C	0.4454 [0.23; 0.69]	0.0111 [0.00; 0.04]	0.0364 [0.00; 0.11]	0.5071 [0.26; 0.73]
G	0.0164 [0.00; 0.07]	0.0147 [0.00; 0.06]	0.1294 [0.05; 0.25]	0.8395 [0.71; 0.93]
T	0.3319 [0.17; 0.48]	0.2020 [0.11; 0.31]	0.2660 [0.14; 0.43]	0.2001 [0.07; 0.34]

Shown are mean estimates of 100.000 MCMC iterations, discarding the first 20.000 iterations as the burn-in, and the corresponding 95% credibility intervals. Despite the wide 95% credibility intervals, the estimates show why such a first-order dependence is useful. Based on the mean estimates, many differences with the estimates of the atpB dataset, shown in Table 2, can be seen.

## Discussion

While nucleotide context-dependent models can offer large improvements in terms of model fit to the data as compared to independent evolutionary models, their performance has to be compared to the performance of both codon-based and codon partition (CP) models. Shapiro et al. [1] state that few analyses in the literature on protein-coding sequences actually take into account the genetic code. Moreover, commonly employed model selection techniques (such as Modeltest by Posada and Crandall [2]) exclude codon-based models from the model comparisons, presumably due to the associated computational cost. Shapiro et al. [1] show, in alignments of 177 RNA virus genes and 106 yeast genes, that the codon-based GY94 model [10] is the model of choice for all the yeast genes and for 67% of the virus genes. However, due the computational cost the GY94 model seems only of use in smaller datasets. Compared to the independence models (such as the frequently used GTR + Γ + I model), the CP models performed better on average for all the alignments analyzed. Such biologically motivated CP models are hence a computationally feasible alternative to codon-based models for use with protein-coding sequences, frequently outperforming standard nucleotide models.

We have shown in this paper that the computational requirements for integrating full codon models in a thermodynamic integration framework for model comparison increase drastically. While we have focused on the model of Goldman and Yang [10] as the full codon model of choice in this paper, better performing codon models have been developed (we refer to [28] for a review on codon substitution models). A study on full codon models is however beyond the scope of this paper, as the main goal was to show that current codon partition models can be approved upon at a computational cost below that of full codon models.

We have shown in this work that codon partition models that are extended with a context-dependence pattern for the third codon position across the entire underlying phylogenetic tree (so-called context-dependent codon partition models) significantly improve model fit compared to traditional codon partition models. While this work was mainly inspired by empirical findings [11], other context-dependent assumptions to extend traditional codon partition models can be devised and tested, which is the subject of ongoing work. The models proposed in this paper illustrate the complex (context-dependent) substitution patterns at the third codon position, along with increased rates of evolution at that position.

The approach we have taken to model context-dependent evolution at the third codon position selects one of 16 models using the neighbouring base combination at the start of each branch, which means that for a given site the context might change at each internal node of the underlying phylogenetic tree. A limiting aspect of this approach however is that the neighbouring base combination is assumed not to change along the length of a branch. This leads to only one in three codon positions (i.e. the third codon position and its two immediate neighbours) that can undergo substitutions, which is an assumption similar to that of full codon models (i.e. only one position in the codon can undergo substitutions). Contrary to the original context-dependent model developed for non-coding sequences [13], the evolution of the third codon position could be allowed to depend upon the neighbouring base combination at both the start and the end of each branch. However, such an assumption leads to 256 possible combinations of neighbours, instead of 16 used in this paper. It therefore seems highly unlikely that a sufficient performance increase is obtained to overthrow the penalty in model fit for the additional parameters. We have hence refrained from testing such an assumption in this paper.

Apart from drastically increasing the number of parameters to model the evolution at the third codon position more closely, as indicated in the previous paragraph, a continuous-time approximation is often used to allow the neighbouring base combinations to evolve along the length of a branch. The approach we've taken to do this partitions each branch into parts with length no greater than 0.005 [29]. As we have shown for non-coding sequences [17], this approach does not yield significantly differing substitution parameter estimates or ancestral root distribution estimates. However, such an approach does lead to higher log Bayes factors compared to when the branches are not split into parts. Given that this approach does not lead to significantly different parameter estimates, the increased log Bayes factor can be attributed to the more accurate approximation of the context-dependent Markov substitution process by allowing the ancestral sequences to change in between the internal speciation nodes [17].

The results shown in this paper hence support the notion that four-fold degenerate sites in the atpB and rbcL protein-coding genes of land plants have a substitution process that is dependent on the composition of its neighbouring bases. In the work that inspired this paper, Morton [11] suggested that a plausible explanation for such context dependence lies with misincorporations by either the DNA polymerase or the mismatch repair process. We refer to different papers by Morton [11,30] and Morton et al. [31,32] for additional plausible explanations for context dependence in the chloroplast genome and to the paper of Hawk et al. [33] for a study on the variation in the efficiency of DNA mismatch repair at different sites in the yeast genome.

Even though we have found that the same context-dependent codon partition model performed best amongst all considered competing models for both atpB and rbcL genes, we have shown that the (context-dependent) substitution patterns at the third codon position differ greatly between both datasets. This means that, should both genes be concatenated to form a larger alignment, different context-dependent codon partition models may need to be used for each gene to perform accurate phylogenetic reconstruction for such a concatenated alignment. The substitution patterns discussed in this paper hence provide additional indications that great care needs to be taken in the analysis of concatenated genes.

As we have introduced the concept of context-dependent codon partition models in this paper, we have not yet undertaken an attempt to perform phylogenetic inference on protein-coding sequences using these models as the relationship between (increases in) model fit and the ability to accurately reconstruct phylogenetic trees is intricately complex [9]. In order to perform an extensive phylogenetic study on the context-dependent codon partition models we have introduced in this paper, additional context-dependent codon partition models need to be programmed and compared against the models presented in this paper, which is the subject of ongoing work. Indeed, while this paper introduces a class of context-dependent codon partition models, only one type of such models is discussed (i.e. those where the evolution of the third codon position depends upon its two immediate neighbours). However, a whole range of such models can be developed, which may for example be aimed towards mimicking full codon models more closely, which was not the aim of this study. The performance of these models can then be compared to the performance of a range of full codon models, in the interest of performing a study on inferring phylogenies. Specifically, the comparison with the codon model of Muse and Gaut [34] would be interesting, since this model, and its derivative MG-type models, relies on estimating only 12 equilibrium frequencies, whereas GY-type models estimate 61 equilibrium codon frequencies [28]. A thorough model comparison, like the one we've performed on non-coding sequences [35], is necessary to determine an accurate evolutionary model for phylogenetic inference.

The computational burden of our context-dependent codon partition models is, as for standard CP models, much lower than for actual codon models due to the decrease in number of parameters and the easier computation of eigenvalues and eigenvectors of the substitution matrices. Indeed, the spectral decomposition of the codon probability matrix (i.e. model) as well as the computation of its powers is considerably slower than for a nucleotide model. Computational effort in computing the eigenvalues and eigenvectors of a matrix rises as the cube of the number of rows or columns, hence the effort is 61^3^/4^3 ^≈ 3,547 times greater for a codon than for a nucleotide model [36]. Since our best-performing context-dependent codon partition model uses 20 nucleotide models to estimates its parameters, computational efforts of a codon model vis-à-vis this model are to 61^3^/(20×4^3^) ≈ 222 times greater. In other words,

codon partition models (context-dependent or not) replace the high dimensionality of codon models by a series (or combination) of low dimensional matrices, for which spectral decomposition requires much less computational efforts.

The computational differences listed in the above paragraph are theoretical however, since often other estimations (such as data augmentation) need to be performed alongside eigenvalue decompositions, which do not require calculation of new eigenvalues. We have hence measured the computation time for the different classes of model present in this paper and have listed the results in Table 5. Given the many different calculations we had to perform, two different systems were used to obtain the results. The architecture used to perform the thermodynamic integration for the codon partition models (CP), context-dependent codon partition models (CDCP) and GY94 model with equal rates is a 4-core AMD Opteron 2356 processor (B3 stepping) clocked at 2.3 Ghz, which has 2 Mb L3 cache, requires DDR2 memory and was introduced in April 2008. The architecture used for the GY94 model with among-codon rate variation is a 8-core Intel Xeon X7560 clocked at 2.27 Ghz, which has 24 Mb L3 cache, requires DDR3 memory and was introduced in March 2010. Note that our software routines are single-threaded. Given the methodology we presented in this paper, it can be concluded that in the thermodynamic integration framework the CDCP models are roughly 2 times more demanding than the existing CP models, whereas the GY94 model with equal rates requires roughly 26 times the computational demands of the existing CP models. The comparison with the GY94 model with among-codon rate variation is more difficult since a different processor architecture was used for those calculations.

**Table 5:** Computational demands: number of iterations and computation time for the comparison of the different models in the thermodynamic integration framework.

Model	Iterations	System	Time	Time (corr.)
CP	1.020.000	4-core Opteron	7d 23 h	-
CDCP	1.050.000	4-core Opteron	16d 7 h	-
GY94	300.000	4-core Opteron	57d 21 h	203 days
GY94+Γ	150.000	8-core Xeon	29d 21 h	210 days

The first column shows the type of model being considered: CP (codon partition), CDCP (context-dependent codon partition), with the most parameter-rich in each of these classes being chosen to provide the computational estimates. The second column contains the number of Bayesian MCMC iterations for the comparison of each class of models vis-à-vis the GTR model in the thermodynamic integration framework. Given the increased computational demands, fewer iterations were run for the GY94 model, which is reflected in the annealing and melting estimates being further apart (see Results section). The third column lists the computer architecture on which the calculations were run. The fourth column contains the computation time for the model comparisons if they would be executed on a single processor core. Finally, the fifth column provides an estimate of the computation time required for the GY94 model should an equal amount of iterations be run as for the CP and CDCP models.

## Conclusions

Designing accurate context-dependent models is a complex process, with many different assumptions that require testing using an accurate procedure for model testing, which is computationally very demanding. In this paper, we show that current codon partition models may benefit from allowing the evolution of four-fold degenerate sites to depend upon the two immediate neighboring sites. Hence, the models we present here do not simply present an intermediate step between codon partition models and full codon models, given that the dependency patterns studied transcend the codon boundaries. Hence, dependencies are inferred that cannot be inferred even from high-dimensional full codon models.

Our analyses of the atpB and rbcL coding regions of a dataset of land plants show that the context-dependent codon partition models presented in this paper significantly outperform current codon partition models. Further, even though for both datasets the same model is selected as yielding the highest increase in model fit, the parameter estimates indicate different substitution patterns in these datasets from a context-dependent point of view. Additional datasets will need to be analyzed to further study these substitution patterns and the way they differ among protein-coding genes.

## Authors' contributions

GB initiated the study, designed the context-dependent codon partition models and the different ancestral root distributions accompanying these models, performed all the analyses, programmed the software routines and wrote a first complete version of the manuscript. YVdP contributed biological expertise to the analyses. SV contributed statistical expertise to the analyses and edited the manuscript. All authors read and approved the final manuscript.

## Supplementary Material

Additional file 1 **Baele et al - Supplementary Material**. File containing supplementary material and information that was not included in the main document. Word 2003 format. Click here for file
