# Supplementary material for: Context-dependent codon partition models provide significant increases in model fit in atpB and rbcL protein-coding genes
Source: BMC Evol Biol. 2011 May 27;11:145. doi: 10.1186/1471-2148-11-145 (PMC3126739; doi:10.1186/1471-2148-11-145)
Supplement: Additional file 1 — Baele et al - Supplementary Material. File containing supplementary material and information that was not included in the main document. Word 2003 format. [file 1471-2148-11-145-S1.DOC]

*atpB dataset – Parameter estimates*

When the left neighbouring base is adenine, the estimates for the rAG, rAC, rAT, rTA, rTG and rTC parameters are not influenced by the identity of the right neighbouring base. When the right neighbouring base is either adenine or cytosine, the rGT estimate is higher than the rGC estimate, whereas these two parameters are nearly equal to one another when the right neighbouring base is guanine or thymine. The rCA and rCG estimates only show a small difference when the right neighbouring base is adenine, but are otherwise not influenced by the identity of the right neighbouring base.

When the left neighbouring base is cytosine, the estimates for the rAC, rAT, rTA, and rTG parameters are not influenced by the identity of the right neighbouring base. The rAG estimate is elevated when the right neighbouring base is guanine, while the rTC estimates increase steadily in the order A < C < G < T. When the right neighbouring base is either adenine or thymine, the rGT estimate is higher than the rGC estimate, whereas these two parameters are nearly equal to one another when the right neighbouring base is cytosine or guanine. Most apparent in terms of context-dependent behaviour is the increasing rCT substitution rate in the order A < C < G < T, as well as the increasing rGA substitution rate in the order T < A < G < C. The rCA and rCG estimates only show a slight difference when the right neighbouring base is adenine, but are otherwise not influenced by the identity of the right neighbouring base.

When the left neighbouring base is guanine, the estimates for the rAG, rAC, rAT, rTA, rTG and rTC parameters are not influenced by the identity of the right neighbouring base. Small differences can be seen between the rGT and rGC estimates, while the major differences can again be found in the rCT and rGA estimates. While the rCT estimate drops when the right neighbouring base is cytosine, the rGA estimate drops when the right neighbouring base is thymine. The rCA and rCG estimates are nearly equal to one another in all contexts, but are slightly lower when the right neighbouring base is guanine.

Finally, when the left neighbouring base is thymine, the estimates for the rAC, rTA, rTG and rTC parameters are not influenced by the identity of the right neighbouring base. The rAT estimate is slightly elevated when the right neighbouring base is adenine, while the rAG estimate is elevated when the right neighbouring base is either cytosine or guanine. While the rGT and rGC estimates are nearly identical when the right neighbouring base is adenine, there is an increasing discrepancy in these estimates in favour of rGT in the order G < C < T. The rCA and rCG estimates are nearly equal to one another when the right neighbouring base is either adenine, cytosine or guanine. When the right neighbouring base is thymine, the rCA and rCG estimates show the largest difference across all contexts, with a clearly elevated rCA estimate. The differences in context-dependent substitution rates discussed here illustrate that the relationship between these substitution rates and the immediate neighbouring bases extends well beyond an influence of the A+T context of those bases, as proposed by Morton .

*rbcL dataset – Parameter estimates*

When the left neighbouring base is adenine, the estimates for the rAG, rAC, rAT, rCA, rCG, rTA, rTG and rTC parameters are not influenced by the identity of the right neighbouring base. The differences for the other parameter estimates are also rather small, such as the increase in the rGA parameter when the right neighbouring base is thymine or the increase in the rGC parameter when the right neighbouring base is adenine or the increase in the rGT parameter when the right neighbouring base is cytosine. There is a noticeable difference between the rGC and rGT estimates when the right neighbouring is adenine, whereas for the other right neighbouring bases, this difference is quite small.

When the left neighbouring base is cytosine, the estimates for the rAC, rCT, rCA, rCG, rTA, rTC and rTG parameters are not influenced by the identity of the right neighbouring base. The rAG estimate is elevated when the right neighbouring base is cytosine, while in that case the rAT and rGT estimates are slightly decreased and the rGA estimate increases drastically. The rGC estimate is the highest when the right neighbouring base is adenine.

When the left neighbouring base is guanine, the estimates for the rAC, rAT, rCA, rCG, rTA, rTG and rTC parameters are not influenced by the identity of the right neighbouring base. The rAG estimates are slightly elevated when the right neighbouring base is either cytosine or guanine. The rCT estimates increase slightly in the order A < C < G < T whereas the rGC estimates increase slightly in the order G < C < T < A. The rGT estimate is drastically higher when the right neighbouring base is adenine and the rGA estimate is elevated when the right neighbouring base is either cytosine or guanine.

Finally, when the left neighbouring base is thymine, the estimates for the rAC, rCT, rCA, rCG, rTG and rTC parameters are not influenced by the identity of the right neighbouring base. The rAG estimate is slightly decreased when the right neighbouring base is thymine, while the rAT estimate is slightly decreased when the right neighbouring base is guanine, the rGT estimate is elevated when the right neighbouring base is guanine and the rGA estimate is decreased when the right neighbouring base is thymine. In other words, for the rbcL datasets as well, these differences in context-dependent substitution rates discussed here illustrate that the relationship between these substitution rates and the immediate neighbouring bases extends well beyond an influence of the A+T context of those bases, as proposed by Morton .

**Table S1 - atpB dataset: Independent and context-dependent models evaluated against the independent GTR model.**

| Model | Annealing | Melting | Log BF |
| --- | --- | --- | --- |
| GTR16C | [270.74 ; 278.07] | [270.82 ; 280.69] | 275.08 |
| GTR_112_ | [354.51 ; 361.86] | [357.05 ; 362.72] | 359.04 |
| GTR_123_ | [548.30 ; 558.03] | [553.08 ; 560.40] | 554.95 |
| GTR_112_+FF_3_ | [656.60 ; 665.04] | [657.84 ; 667.77] | 661.81 |
| GTR_123_+FF_3_ | [864.46 ; 872.93] | [862.79 ; 870.18] | 867.59 |
| GTR_112_+FF_16_ | [621.62 ; 629.82] | [626.02 ; 634.39] | 627.96 |
| GTR_123_+FF_16_ | [825.05 ; 834.00] | [828.51 ; 837.86] | 831.69 |
| GTR_112_+CD_3_ | [384.19 ; 390.48] | [386.81 ; 392.69] | 388.54 |
| GTR_123_+CD_3_ | [586.77 ; 594.50] | [593.42 ; 601.43] | 594.03 |
| GTR_112_+CD_16_ | [486.95 ; 494.35] | [491.34 ; 499.39] | 493.01 |
| GTR_123_+CD_16_ | [689.15 ; 695.96] | [704.33 ; 714.26] | 700.93 |
|  |  |  |  |
| GTR+Γ | [1384.32 ; 1402.99] | [1393.75 ; 1410.96] | 1398.01 |
| GTR16C+Γ | [1572.39 ; 1590.09] | [1581.16 ; 1599.56] | 1585.80 |
| GTR_112_+Γ | [1446.22 ; 1465.20] | [1497.94 ; 1519.71] | 1482.27 |
| GTR_123_+Γ | [1474.64 ; 1493.12] | [1528.11 ; 1550.06] | 1511.48 |
| GTR_112_+FF_3_+Γ | [1752.04 ; 1769.01] | [1763.46 ; 1776.33] | 1765.21 |
| GTR_123_+FF_3_+Γ | [1755.27 ; 1769.56] | [1808.62 ; 1825.73] | 1789.80 |
| GTR_112_+FF_16_+Γ | [1751.41 ; 1767.30] | [1771.22 ; 1789.51] | 1769.86 |
| GTR_123_+FF_16_+Γ | [1757.59 ; 1776.08] | [1793.80 ; 1811.02] | 1784.62 |
| GTR_112_+CD_3_+Γ | [1471.84 ; 1486.25] | [1514.01 ; 1536.59] | 1502.17 |
| GTR_123_+CD_3_+Γ | [1522.02 ; 1541.74] | [1547.34 ; 1571.61] | 1545.68 |
| GTR_112_+CD_16_+Γ | [1599.46 ; 1615.13] | [1609.02 ; 1629.82] | 1613.36 |
| GTR_123_+CD_16_+Γ | [1624.31 ; 1641.09] | [1652.58 ; 1670.22] | 1647.05 |
|  |  |  |  |
| GTR_112_+Γ_112_ | [1729.97 ; 1755.76] | [1768.48 ; 1802.65] | 1764.22 |
| GTR_123_+Γ_123_ | [1694.40 ; 1717.93] | [1754.15 ; 1783.63] | 1737.53 |
| GTR_112_+FF_3_+Γ_112_ | [2041.39 ; 2062.11] | [2049.64 ; 2071.56] | 2056.17 |
| GTR_123_+FF_3_+Γ_123_ | [2033.71 ; 2053.26] | [2052.07 ; 2071.58] | 2052.66 |
| GTR_112_+FF_16_+Γ_112_ | [2028.66 ; 2050.93] | [2053.63 ; 2083.15] | 2054.09 |
| GTR_123_+FF_16_+Γ_123_ | [2009.27 ; 2030.80] | [2035.99 ; 2062.81] | 2034.72 |
| GTR_112_+CD_3_+Γ_112_ | [1778.17 ; 1805.21] | [1763.90 ; 1788.34] | 1783.90 |
| GTR_123_+CD_3_+Γ_123_ | [1737.22 ; 1755.13] | [1782.41 ; 1813.69] | 1772.11 |
| GTR_112_+CD_16_+Γ_112_ | [1882.82 ; 1903.83] | [1903.62 ; 1930.73] | 1905.25 |
| GTR_123_+CD_16_+Γ_123_ | [1878.57 ; 1898.48] | [1911.61 ; 1938.21] | 1906.72 |

The following series of evolutionary models were evaluated against the independent GTR model: models without the assumption of among-site rate variation (models 1 through 11; i.e. GTR16C through GTR_123_+CD_16_), models with the assumption of a single among-site rate variation distribution for all sites in the alignment (models 12 through 23; i.e. GTR+Γ through GTR_123_+CD_16_+Γ) and models with the assumption of an among-site rate variation distribution per codon position (models 24 through 33; i.e. GTR_112_+Γ_112_ through GTR_123_+CD_16_+Γ_123_). The first column contains the model notation; the second and third column contain the 95% confidence interval for the log Bayes Factor calculated using the annealing and melting schemes of the thermodynamic integration approach; the fourth column contains the bidirectional mean log Bayes Factor for the model comparison.

**Table S2 – atpB dataset: Independent and context-dependent models evaluated against the independent GTR model (continued).**

| Model | Annealing | Melting | Log BF |
| --- | --- | --- | --- |
| GTR_112_+Γ_112_+CP_112_ | [1968.15 ; 1993.60] | [1999.15 ; 2031.13] | 1998.01 |
| GTR_123_+Γ_123_+CP_123_ | [1994.84 ; 2018.60] | [2023.93 ; 2052.01] | 2022.34 |
| GTR_112_+FF_3_+Γ_112_+CP_112_ | [2271.80 ; 2292.08] | [2290.17 ; 2314.91] | 2292.24 |
| GTR_123_+FF_3_+Γ_123_+CP_123_ | [2290.09 ; 2307.44] | [2306.47 ; 2330.69] | 2308.67 |
| GTR_112_+FF_16_+Γ_112_+CP_112_ | [2257.18 ; 2276.19] | [2279.34 ; 2302.47] | 2278.79 |
| GTR_123_+FF_16_+Γ_123_+CP_123_ | [2284.30 ; 2302.61] | [2307.29 ; 2327.37] | 2305.39 |
| GTR_112_+CD_3_+Γ_112_+CP_112_ | [1992.47 ; 2016.74] | [2010.74 ; 2042.41] | 2015.59 |
| GTR_123_+CD_3_+Γ_123_+CP_123_ | [2016.97 ; 2035.57] | [2030.44 ; 2059.30] | 2035.57 |
| GTR_112_+CD_16_+Γ_112_+CP_112_ | [2104.57 ; 2125.24] | [2120.74 ; 2138.63] | 2122.29 |
| GTR_123_+CD_16_+Γ_123_+CP_123_ | [2122.52 ; 2138.93] | [2137.19 ; 2152.97] | 2137.90 |
|  |  |  |  |
| GTR_112_+Γ_112_+CP_112_+F_112_ | [2242.38 ; 2272.68] | [2257.72 ; 2338.60] | 2277.84 |
| GTR_123_+Γ_123_+CP_123_+F_123_ | [2278.64 ; 2302.60] | [2316.58 ; 2361.11] | 2314.73 |
| GTR_112_+FF_3_+Γ_112_+CP_112_+F_112_ | [2527.83 ; 2555.73] | [2545.57 ; 2576.19] | 2551.33 |
| GTR_123_+FF_3_+Γ_123_+CP_123_+F_123_ | [2572.32 ; 2601.70] | [2586.06 ; 2612.23] | 2593.08 |
| GTR_112_+FF_16_+Γ_112_+CP_112_+F_112_ | [2525.98 ; 2553.82] | [2528.59 ; 2560.45] | 2542.21 |
| GTR_123_+FF_16_+Γ_123_+CP_123_+F_123_ | [2554.38 ; 2583.37] | [2569.80 ; 2619.27] | 2581.70 |
| GTR_112_+CD_3_+Γ_112_+CP_112_+F_112_ | [2257.84 ; 2285.09] | [2284.24 ; 2315.02] | 2285.54 |
| GTR_123_+CD_3_+Γ_123_+CP_123_+F_123_ | [2295.07 ; 2325.02] | [2306.07 ; 2337.26] | 2315.86 |
| GTR_112_+CD_16_+Γ_112_+CP_112_+F_112_ | [2358.43 ; 2385.93] | [2368.56 ; 2403.39] | 2379.08 |
| GTR_123_+CD_16_+Γ_123_+CP_123_+F_123_ | [2388.10 ; 2412.92] | [2400.32 ; 2427.59] | 2407.23 |

The following series of evolutionary models were evaluated against the independent GTR model: models with the assumption of an among-site rate variation distribution, with possible different mean rates, per codon position (models 1 through 10; i.e. GTR_112_+Γ_112_+CP_112_ through GTR_123_+CD_16_+Γ_123_+CP_123_) and models with the assumption of an among-site rate variation distribution, with possible different mean rates, per codon position and accommodating the nucleotide frequency bias (models 11 through 20; i.e. GTR_112_+Γ_112_+CP_112_+F_112_ through GTR_123_+CD_16_+Γ_123_+CP_123_+F_123_). The first column contains the model notation; the second and third column contain the 95% confidence interval for the log Bayes Factor calculated using the annealing and melting schemes of the thermodynamic integration approach; the fourth column contains the bidirectional mean log Bayes Factor for the model comparison.

**Table S3 – atpB dataset: Independent and context-dependent models evaluated against the independent GTR model (concluding).**

| Model | Annealing | Melting | Log BF |
| --- | --- | --- | --- |
| GTR_112_+FF_3_+Γ_112_+CP_112_+F_112_+3Z | [2508.57 ; 2543.42] | [2561.13 ; 2599.72] | 2553.21 |
| GTR_123_+FF_3_+Γ_123_+CP_123_+F_123_+3Z | [2564.52 ; 2594.59] | [2587.61 ; 2625.98] | 2593.17 |
| GTR_112_+FF_16_+Γ_112_+CP_112_+F_112_+3Z | [2499.97 ; 2538.13] | [2554.32 ; 2592.05] | 2546.12 |
| GTR_123_+FF_16_+Γ_123_+CP_123_+F_123_+3Z | [2540.87 ; 2572.51] | [2594.42 ; 2633.29] | 2585.27 |
| GTR_112_+CD_3_+Γ_112_+CP_112_+F_112_+3Z | [2267.58 ; 2309.07] | [2291.16 ; 2358.35] | 2306.54 |
| GTR_123_+CD_3_+Γ_123_+CP_123_+F_123_+3Z | [2307.59 ; 2347.95] | [2328.01 ; 2372.80] | 2339.09 |
| GTR_112_+CD_16_+Γ_112_+CP_112_+F_112_+3Z | [2318.54 ; 2362.36] | [2424.12 ; 2470.74] | 2393.94 |
| GTR_123_+CD_16_+Γ_123_+CP_123_+F_123_+3Z | [2405.39 ; 2433.69] | [2433.33 ; 2462.78] | 2433.80 |
|  |  |  |  |
| GTR_112_+FF_3_+Γ_112_+CP_112_+F_112_+3F | [2485.47 ; 2523.38] | [2580.51 ; 2622.50] | 2552.97 |
| GTR_123_+FF_3_+Γ_123_+CP_123_+F_123_+3F | [2575.38 ; 2605.15] | [2604.76 ; 2647.55] | 2608.21 |
| GTR_112_+FF_16_+Γ_112_+CP_112_+F_112_+3F | [2478.32 ; 2512.29] | [2576.86 ; 2618.39] | 2546.47 |
| GTR_123_+FF_16_+Γ_123_+CP_123_+F_123_+3F | [2548.88 ; 2578.45] | [2595.11 ; 2638.76] | 2590.30 |
| GTR_112_+CD_3_+Γ_112_+CP_112_+F_112_+3F | [2313.43 ; 2352.33] | [2351.19 ; 2395.42] | 2353.09 |
| GTR_123_+CD_3_+Γ_123_+CP_123_+F_123_+3F | [2348.37 ; 2380.19] | [2365.48 ; 2404.65] | 2374.67 |
| GTR_112_+CD_16_+Γ_112_+CP_112_+F_112_+3F | [2365.61 ; 2407.62] | [2404.69 ; 2439.28] | 2404.30 |
| GTR_123_+CD_16_+Γ_123_+CP_123_+F_123_+3F | [2418.55 ; 2446.09] | [2438.06 ; 2468.00] | 2442.68 |
|  |  |  |  |
| GTR_112_+FF_3_+Γ_112_+CP_112_+F_112_+3S | [2475.08 ; 2511.85] | [2588.19 ; 2626.18] | 2550.32 |
| GTR_123_+FF_3_+Γ_123_+CP_123_+F_123_+3S | [2554.90 ; 2585.93] | [2594.95 ; 2635.56] | 2592.83 |
| GTR_112_+FF_16_+Γ_112_+CP_112_+F_112_+3S | [2461.37 ; 2493.34] | [2570.77 ; 2613.95] | 2534.86 |
| GTR_123_+FF_16_+Γ_123_+CP_123_+F_123_+3S | [2541.92 ; 2572.93] | [2590.12 ; 2633.83] | 2584.70 |
| GTR_112_+CD_3_+Γ_112_+CP_112_+F_112_+3S | [2286.41 ; 2324.46] | [2318.25 ; 2362.26] | 2322.84 |
| GTR_123_+CD_3_+Γ_123_+CP_123_+F_123_+3S | [2328.82 ; 2360.01] | [2357.01 ; 2390.48] | 2359.08 |
| GTR_112_+CD_16_+Γ_112_+CP_112_+F_112_+3S | [2352.17 ; 2387.35] | [2412.19 ; 2448.66] | 2400.09 |
| GTR_123_+CD_16_+Γ_123_+CP_123_+F_123_+3S | [2405.93 ; 2447.09] | [2429.95 ; 2466.85] | 2437.46 |

The following series of evolutionary models were evaluated against the independent GTR model: models with the assumption of an among-site rate variation distribution, with possible different mean rates, per codon position, accommodating nucleotide frequency bias and assuming an independent ancestral root distribution for the third codon position (models 1 through 8; i.e. GTR_112_+FF_3_+Γ_112_+CP_112_+F_112_+3Z through GTR_123_+CD_16_+Γ_123_+CP_123_+F_123_+3Z), a first-order Markov chain as the ancestral root distribution at the third codon position (models 9 through 16; i.e. GTR_112_+FF_3_+Γ_112_+CP_112_+F_112_+3F through GTR_123_+CD_16_+Γ_123_+CP_123_+F_123_+3F) and a dependency of the ancestral third codon position upon its two immediate neighbours (models 17 through 24; i.e. GTR_112_+FF_3_+Γ_112_+CP_112_+F_112_+3S through GTR_123_+CD_16_+Γ_123_+CP_123_+F_123_+3S). The first column contains the model notation; the second and third column contain the 95% confidence interval for the log Bayes Factor calculated using the annealing and melting schemes of the thermodynamic integration approach; the fourth column contains the bidirectional mean log Bayes Factor for the model comparison.

**Table S4 - rbcL dataset: Independent and context-dependent models evaluated against the independent GTR model.**

| Model | Annealing | Melting | Log BF |
| --- | --- | --- | --- |
| GTR16C | [318.43 ; 324.09] | [320.53 ; 329.63] | 323.17 |
| GTR_112_ | [255.02 ; 260.94] | [256.88 ; 263.75] | 259.15 |
| GTR_123_ | [399.91 ; 406.66] | [399.89 ; 407.82] | 403.57 |
| GTR_112_+FF_3_ | [611.94 ; 619.03] | [617.90 ; 625.72] | 618.65 |
| GTR_123_+FF_3_ | [750.85 ; 758.30] | [758.17 ; 765.86] | 758.30 |
| GTR_112_+FF_16_ | [584.43 ; 592.73] | [581.20 ; 591.58] | 587.48 |
| GTR_123_+FF_16_ | [725.23 ; 733.82] | [730.00 ; 736.62] | 731.42 |
| GTR_112_+CD_3_ | [319.76 ; 327.88] | [321.25 ; 327.82] | 324.18 |
| GTR_123_+CD_3_ | [468.23 ; 476.04] | [466.65 ; 473.66] | 471.15 |
| GTR_112_+CD_16_ | [482.04 ; 488.75] | [485.45 ; 494.69] | 487.73 |
| GTR_123_+CD_16_ | [627.25 ; 635.08] | [628.50 ; 637.05] | 631.97 |
|  |  |  |  |
| GTR+Γ | [1094.96 ; 1102.14] | [1109.92 ; 1117.22] | 1106.06 |
| GTR16C+Γ | [1366.37 ; 1378.66] | [1384.11 ; 1397.96] | 1381.77 |
| GTR_112_+Γ | [1231.65 ; 1242.86] | [1256.19 ; 1268.48] | 1249.79 |
| GTR_123_+Γ | [1252.55 ; 1263.46] | [1263.67 ; 1276.58] | 1264.07 |
| GTR_112_+FF_3_+Γ | [1581.40 ; 1593.92] | [1612.93 ; 1629.92] | 1604.54 |
| GTR_123_+FF_3_+Γ | [1601.14 ; 1614.15] | [1616.55 ; 1630.24] | 1615.52 |
| GTR_112_+FF_16_+Γ | [1572.89 ; 1584.79] | [1586.14 ; 1600.03] | 1586.46 |
| GTR_123_+FF_16_+Γ | [1586.00 ; 1598.16] | [1610.13 ; 1624.85] | 1604.79 |
| GTR_112_+CD_3_+Γ | [1306.54 ; 1318.02] | [1313.30 ; 1324.57] | 1315.61 |
| GTR_123_+CD_3_+Γ | [1316.11 ; 1327.36] | [1324.08 ; 1333.97] | 1325.38 |
| GTR_112_+CD_16_+Γ | [1456.45 ; 1467.77] | [1465.65 ; 1479.87] | 1467.44 |
| GTR_123_+CD_16_+Γ | [1475.54 ; 1488.43] | [1477.63 ; 1492.24] | 1483.46 |
|  |  |  |  |
| GTR_112_+Γ_112_ | [1506.29 ; 1520.54] | [1513.02 ; 1528.53] | 1517.10 |
| GTR_123_+Γ_123_ | [1546.19 ; 1559.00] | [1553.10 ; 1567.60] | 1556.47 |
| GTR_112_+FF_3_+Γ_112_ | [1866.93 ; 1882.43] | [1876.51 ; 1893.50] | 1879.84 |
| GTR_123_+FF_3_+Γ_123_ | [1910.06 ; 1925.32] | [1922.90 ; 1940.46] | 1924.69 |
| GTR_112_+FF_16_+Γ_112_ | [1855.68 ; 1869.74] | [1857.05 ; 1874.05] | 1864.13 |
| GTR_123_+FF_16_+Γ_123_ | [1893.88 ; 1909.18] | [1907.90 ; 1924.37] | 1908.83 |
| GTR_112_+CD_3_+Γ_112_ | [1566.92 ; 1580.21] | [1567.71 ; 1585.68] | 1575.13 |
| GTR_123_+CD_3_+Γ_123_ | [1600.90 ; 1614.02] | [1609.41 ; 1622.60] | 1611.74 |
| GTR_112_+CD_16_+Γ_112_ | [1731.07 ; 1745.34] | [1744.72 ; 1759.83] | 1745.24 |
| GTR_123_+CD_16_+Γ_123_ | [1767.43 ; 1782.84] | [1785.69 ; 1801.57] | 1784.38 |

The following series of evolutionary models were evaluated against the independent GTR model: models without the assumption of among-site rate variation (models 1 through 11; i.e. GTR16C through GTR_123_+CD_16_), models with the assumption of a single among-site rate variation distribution for all sites in the alignment (models 12 through 23; i.e. GTR+Γ through GTR_123_+CD_16_+Γ) and models with the assumption of an among-site rate variation distribution per codon position (models 24 through 33; i.e. GTR_112_+Γ_112_ through GTR_123_+CD_16_+Γ_123_). The first column contains the model notation; the second and third column contain the 95% confidence interval for the log Bayes Factor calculated using the annealing and melting schemes of the thermodynamic integration approach; the fourth column contains the bidirectional mean log Bayes Factor for the model comparison.

**Table S5 – rbcL dataset: Independent and context-dependent models evaluated against the independent GTR model (continued).**

| Model | Annealing | Melting | Log BF |
| --- | --- | --- | --- |
| GTR_112_+Γ_112_+CP_112_ | [1674.27 ; 1686.61] | [1679.27 ; 1690.69] | 1682.71 |
| GTR_123_+Γ_123_+CP_123_ | [1686.32 ; 1695.28] | [1690.82 ; 1702.08] | 1693.62 |
| GTR_112_+FF_3_+Γ_112_+CP_112_ | [2044.61 ; 2055.78] | [2049.26 ; 2063.76] | 2053.35 |
| GTR_123_+FF_3_+Γ_123_+CP_123_ | [2066.97 ; 2076.53] | [2072.61 ; 2086.81] | 2075.73 |
| GTR_112_+FF_16_+Γ_112_+CP_112_ | [2037.74 ; 2050.39] | [2038.25 ; 2050.77] | 2044.29 |
| GTR_123_+FF_16_+Γ_123_+CP_123_ | [2049.95 ; 2061.18] | [2064.78 ; 2077.58] | 2063.37 |
| GTR_112_+CD_3_+Γ_112_+CP_112_ | [1722.57 ; 1733.07] | [1736.89 ; 1746.68] | 1734.80 |
| GTR_123_+CD_3_+Γ_123_+CP_123_ | [1746.75 ; 1756.16] | [1754.44 ; 1770.16] | 1756.88 |
| GTR_112_+CD_16_+Γ_112_+CP_112_ | [1898.59 ; 1910.68] | [1907.97 ; 1921.50] | 1909.68 |
| GTR_123_+CD_16_+Γ_123_+CP_123_ | [1915.21 ; 1924.76] | [1918.95 ; 1933.03] | 1922.98 |
|  |  |  |  |
| GTR_112_+Γ_112_+CP_112_+F_112_ | [1803.75 ; 1872.68] | [1839.90 ; 1870.07] | 1835.35 |
| GTR_123_+Γ_123_+CP_123_+F_123_ | [1835.66 ; 1854.31] | [1833.46 ; 1856.40] | 1844.96 |
| GTR_112_+FF_3_+Γ_112_+CP_112_+F_112_ | [2154.03 ; 2172.72] | [2162.52 ; 2181.65] | 2167.73 |
| GTR_123_+FF_3_+Γ_123_+CP_123_+F_123_ | [2186.79 ; 2204.68] | [2191.02 ; 2234.51] | 2204.25 |
| GTR_112_+FF_16_+Γ_112_+CP_112_+F_112_ | [2137.21 ; 2158.78] | [2138.79 ; 2169.89] | 2151.17 |
| GTR_123_+FF_16_+Γ_123_+CP_123_+F_123_ | [2166.22 ; 2182.97] | [2177.06 ; 2194.70] | 2180.24 |
| GTR_112_+CD_3_+Γ_112_+CP_112_+F_112_ | [1829.98 ; 1852.39] | [1852.65 ; 1873.22] | 1852.06 |
| GTR_123_+CD_3_+Γ_123_+CP_123_+F_123_ | [1875.69 ; 1894.25] | [1874.14 ; 1892.45] | 1884.13 |
| GTR_112_+CD_16_+Γ_112_+CP_112_+F_112_ | [1993.96 ; 2013.22] | [2003.68 ; 2028.57] | 2009.86 |
| GTR_123_+CD_16_+Γ_123_+CP_123_+F_123_ | [2026.28 ; 2044.19] | [2032.58 ; 2052.11] | 2038.79 |

The following series of evolutionary models were evaluated against the independent GTR model: models with the assumption of an among-site rate variation distribution, with possible different mean rates, per codon position (models 1 through 10; i.e. GTR_112_+Γ_112_+CP_112_ through GTR_123_+CD_16_+Γ_123_+CP_123_) and models with the assumption of an among-site rate variation distribution, with possible different mean rates, per codon position and accommodating the nucleotide frequency bias (models 11 through 20; i.e. GTR_112_+Γ_112_+CP_112_+F_112_ through GTR_123_+CD_16_+Γ_123_+CP_123_+F_123_). The first column contains the model notation; the second and third column contain the 95% confidence interval for the log Bayes Factor calculated using the annealing and melting schemes of the thermodynamic integration approach; the fourth column contains the bidirectional mean log Bayes Factor for the model comparison.

**Table S6 – rbcL dataset: Independent and context-dependent models evaluated against the independent GTR model (concluding).**

| Model | Annealing | Melting | Log BF |
| --- | --- | --- | --- |
| GTR_112_+FF_3_+Γ_112_+CP_112_+F_112_+3Z | [2185.10 ; 2213.43] | [2201.83 ; 2228.60] | 2207.24 |
| GTR_123_+FF_3_+Γ_123_+CP_123_+F_123_+3Z | [2224.44 ; 2256.44] | [2229.44 ; 2253.52] | 2240.96 |
| GTR_112_+FF_16_+Γ_112_+CP_112_+F_112_+3Z | [2177.02 ; 2209.05] | [2182.37 ; 2208.47] | 2194.22 |
| GTR_123_+FF_16_+Γ_123_+CP_123_+F_123_+3Z | [2199.64 ; 2223.16] | [2210.66 ; 2233.56] | 2216.76 |
| GTR_112_+CD_3_+Γ_112_+CP_112_+F_112_+3Z | [1894.26 ; 1921.09] | [1933.15 ; 1963.99] | 1928.12 |
| GTR_123_+CD_3_+Γ_123_+CP_123_+F_123_+3Z | [1914.95 ; 1942.68] | [1982.80 ; 2016.90] | 1964.33 |
| GTR_112_+CD_16_+Γ_112_+CP_112_+F_112_+3Z | [1991.42 ; 2037.89] | [2012.36 ; 2037.61] | 2019.82 |
| GTR_123_+CD_16_+Γ_123_+CP_123_+F_123_+3Z | [2031.95 ; 2063.50] | [2039.30 ; 2059.54] | 2048.57 |
|  |  |  |  |
| GTR_112_+FF_3_+Γ_112_+CP_112_+F_112_+3F | [2235.57 ; 2260.55] | [2243.85 ; 2271.17] | 2252.78 |
| GTR_123_+FF_3_+Γ_123_+CP_123_+F_123_+3F | [2254.25 ; 2282.48] | [2270.45 ; 2299.40] | 2276.65 |
| GTR_112_+FF_16_+Γ_112_+CP_112_+F_112_+3F | [2215.41 ; 2239.67] | [2234.19 ; 2263.68] | 2238.24 |
| GTR_123_+FF_16_+Γ_123_+CP_123_+F_123_+3F | [2243.88 ; 2277.90] | [2266.62 ; 2295.82] | 2271.06 |
| GTR_112_+CD_3_+Γ_112_+CP_112_+F_112_+3F | [1970.16 ; 2002.40] | [1989.36 ; 2030.26] | 1998.05 |
| GTR_123_+CD_3_+Γ_123_+CP_123_+F_123_+3F | [2006.21 ; 2030.79] | [2020.73 ; 2051.18] | 2027.23 |
| GTR_112_+CD_16_+Γ_112_+CP_112_+F_112_+3F | [2073.329 ; 2096.98] | [2077.55 ; 2109.36] | 2089.30 |
| GTR_123_+CD_16_+Γ_123_+CP_123_+F_123_+3F | [2100.86 ; 2123.32] | [2118.08 ; 2147.39] | 2122.41 |
|  |  |  |  |
| GTR_112_+FF_3_+Γ_112_+CP_112_+F_112_+3S | [2216.06 ; 2241.74] | [2225.54 ; 2254.75] | 2234.52 |
| GTR_123_+FF_3_+Γ_123_+CP_123_+F_123_+3S | [2244.09 ; 2278.18] | [2259.15 ; 2302.28] | 2270.93 |
| GTR_112_+FF_16_+Γ_112_+CP_112_+F_112_+3S | [2188.50 ; 2224.91] | [2208.59 ; 2236.49] | 2214.62 |
| GTR_123_+FF_16_+Γ_123_+CP_123_+F_123_+3S | [2216.84 ; 2247.24] | [2226.87 ; 2273.17] | 2241.03 |
| GTR_112_+CD_3_+Γ_112_+CP_112_+F_112_+3S | [1944.38 ; 1982.91] | [1961.63 ; 1988.06] | 1969.25 |
| GTR_123_+CD_3_+Γ_123_+CP_123_+F_123_+3S | [1976.05 ; 2006.17] | [1983.96 ; 2008.34] | 1993.63 |
| GTR_112_+CD_16_+Γ_112_+CP_112_+F_112_+3S | [2056.52 ; 2077.77] | [2056.90 ; 2079.79] | 2067.74 |
| GTR_123_+CD_16_+Γ_123_+CP_123_+F_123_+3S | [2079.77 ; 2108.70] | [2092.19 ; 2115.89] | 2099.14 |

The following series of evolutionary models were evaluated against the independent GTR model: models with the assumption of an among-site rate variation distribution, with possible different mean rates, per codon position, accommodating nucleotide frequency bias and assuming an independent ancestral root distribution for the third codon position (models 1 through 8; i.e. GTR_112_+FF_3_+Γ_112_+CP_112_+F_112_+3Z through GTR_123_+CD_16_+Γ_123_+CP_123_+F_123_+3Z), a first-order Markov chain as the ancestral root distribution at the third codon position (models 9 through 16; i.e. GTR_112_+FF_3_+Γ_112_+CP_112_+F_112_+3F through GTR_123_+CD_16_+Γ_123_+CP_123_+F_123_+3F) and a dependency of the ancestral third codon position upon its two immediate neighbours (models 17 through 24; i.e. GTR_112_+FF_3_+Γ_112_+CP_112_+F_112_+3S through GTR_123_+CD_16_+Γ_123_+CP_123_+F_123_+3S). The first column contains the model notation; the second and third column contain the 95% confidence interval for the log Bayes Factor calculated using the annealing and melting schemes of the thermodynamic integration approach; the fourth column contains the bidirectional mean log Bayes Factor for the model comparison.
